# A comparative analysis using flowmeter, laser-Doppler |spectrophotometry, and indocyanine green-videoangiography for detection of vascular stenosis in free flaps

**DOI:** 10.1038/s41598-020-57777-2

**Published:** 2020-01-22

**Authors:** Thomas Mücke, Alexander Hapfelmeier, Leonard H. Schmidt, Andreas M. Fichter, Anastasios Kanatas, Klaus-Dietrich Wolff, Lucas M. Ritschl

**Affiliations:** 1 Department of Oral and Maxillofacial Surgery, Malteser Kliniken Rhein-Ruhr, Krefeld-Uerdingen, Germany; 2 Institute of Medical Informatics, Statistics and Epidemiology, School of Medicine, Technical University of Munich, Munich, Germany; 3 Department of Oral and Maxillofacial Surgery, School of Medicine, Technical University of Munich, Klinikum rechts der Isar, Munich, Germany; 4 Leeds Teaching Hospitals, St James Institute of Oncology and Leeds Dental Institute, Munich, Germany

**Keywords:** Experimental models of disease, Surgical oncology

## Abstract

The effects of gradual vascular occlusion on the blood supply of perfused areas are poorly described. Information relating to the comparison of flap monitoring techniques is lacking. Varying stenotic conditions (0%, 25%, 50%, 75% and 100%) were generated on purpose at the A. and V. femoralis in the rat model. Analyses included flowmeter, simultaneous laser-Doppler flowmetry and tissue spectrophotometry (O2C) and indocyanine green- (ICG-) videoangiography with integrated FLOW 800 tool. A Random Forests prediction model was used to analyse the importance of each method to diagnose the stenotic conditions. The ability to discriminate and to accurately estimate the probability of stenosis was assessed by Receiver Operating Characteristic (ROC) curves and calibration plots. Blood flow changes for all modalities were described in detail. Flowmeter displayed earliest a linear decrease as a result of increasing stenosis. A stenosis of 50% degrees was most difficult to detect correctly. The combination of flowmeter and ICG-videoangiography showed high diagnostic power for each stenotic situation (area under the ROC > 0.79). Flowmeter and ICG-videoangiography showed to be most relevant in detection of varying stenotic conditions and may change the clinical outcome. The O2C showed less effect on varying stenotic situations as the only surface monitoring device.

## Introduction

The microvascular free tissue transfer has revolutionised reconstructive microsurgery and has improved the patients’ functional outcome^1^. Despite the well documented co-morbidities of patients undergoing head and neck surgery, free flap surgery has contributed to a continuous improvement in the patient-reported and clinical outcomes^2–4^. Due to the significant patient co-morbidities and the complexity of the operations, this patient cohort has a high free flap failure rate. A successful outcome necessitates a detailed pre-, intra- and postoperative patient screening in addition to the free flap monitoring, for at least the first 72 hours, postoperatively. The reasons for free flap failures are commonly vascular (thrombosis, endothelial damage) or extravascular, (hematoma, kinking, twisting) in origin. Flap necrosis invariably leads to a prolonged hospital stay and may result in mortality due to poor physiological reserves. There is a definitive window of surgical intervention that must not be missed, for the salvage operation to be successful^5,6^. For this reason, the routine postoperative free flap monitoring has to be effective, reproducible and reliable. Free flap monitoring techniques vary and may include the clinical observation, pinprick testing and handheld Doppler^7^. More sophisticated, objective methods include simultaneous laser-Doppler flowmetry and tissue spectrophotometry, tissue oximetry monitoring, infrared thermography and indocyanine green- (ICG-) videoangiography^8–15^.

The changing nature of surgical training, with restricted resident work hours, may affect the continuity of care and render essential the need for close monitoring^16^. Apart from predictable and valid, the monitoring technique must be cost-effective to the clinical team. ICG-videoangiography was introduced into reconstructive free flap surgery in the last 20 years. More recently, this technique was also applied for the immediate analysis of patency following microvascular anastomosis or to predict the incidence of necrosis on the basis of only one measurement as an integrated system in the operating microscope^15,17,18^.

The purpose of this study was to evaluate the outcome of varying stenotic vascular situations, on the local and distal flap perfusion, with three established modalities in a well described rat model^19^.

## Results

### Descriptive analysis

**Figure 1:**
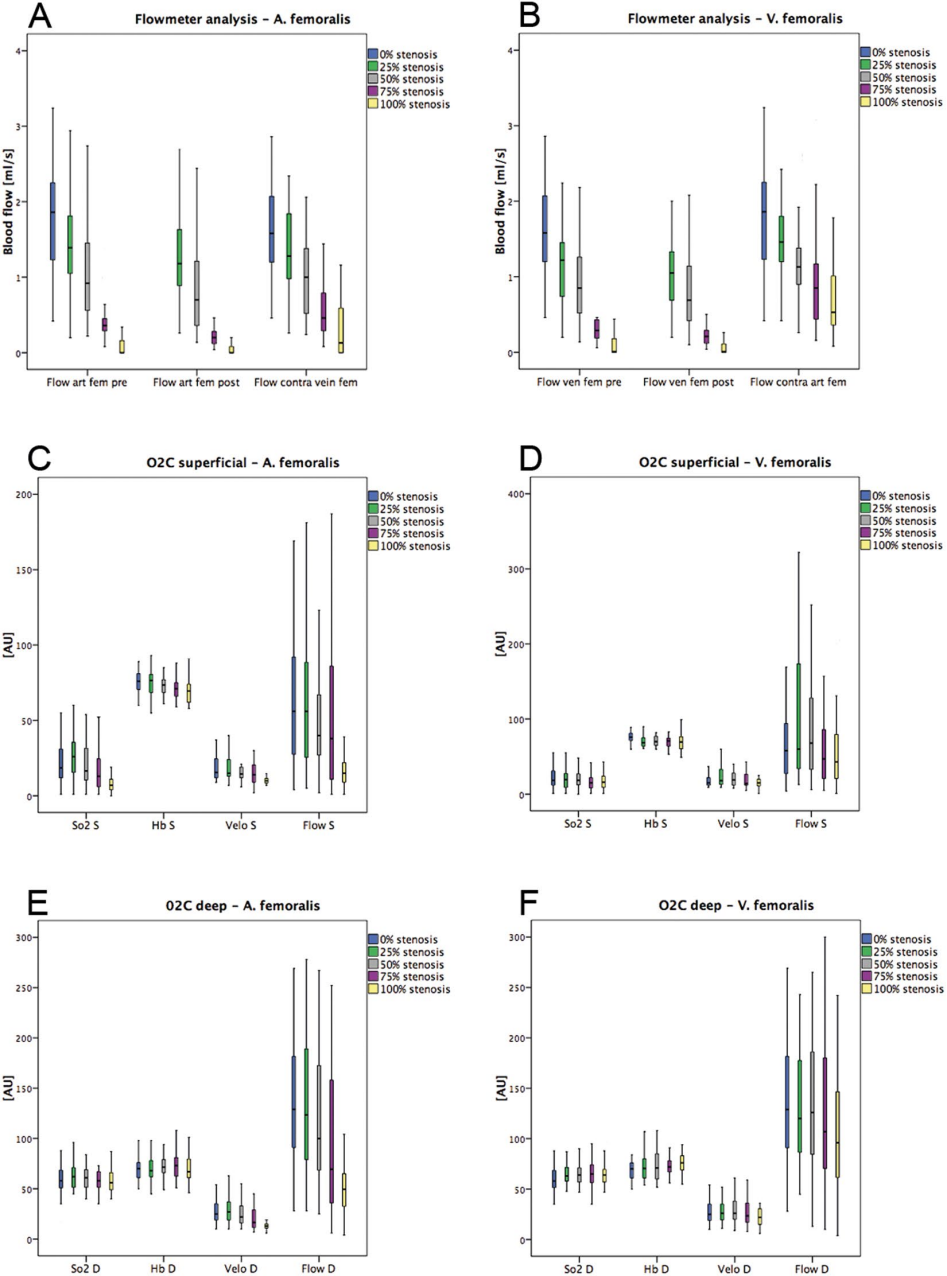
Descriptive results of flowmeter and simultaneous laser-Doppler flowmetry and tissue spectrophotometry (O2C). The results are shown for each modality with varying degrees of arterial (**A,C,E**) or venous (**B,D,F**) stenotic situations. O2C measurements registered superficially at the skin surface (S) and at a depth (D) of 8 mm oxygen saturation (SO_2_, in arbitrary units, [AU]), haemoglobin level (Hb, in [AU]), velocity (velo, in [AU]) and blood flow (flow, in [AU]).

**Figure 2:**
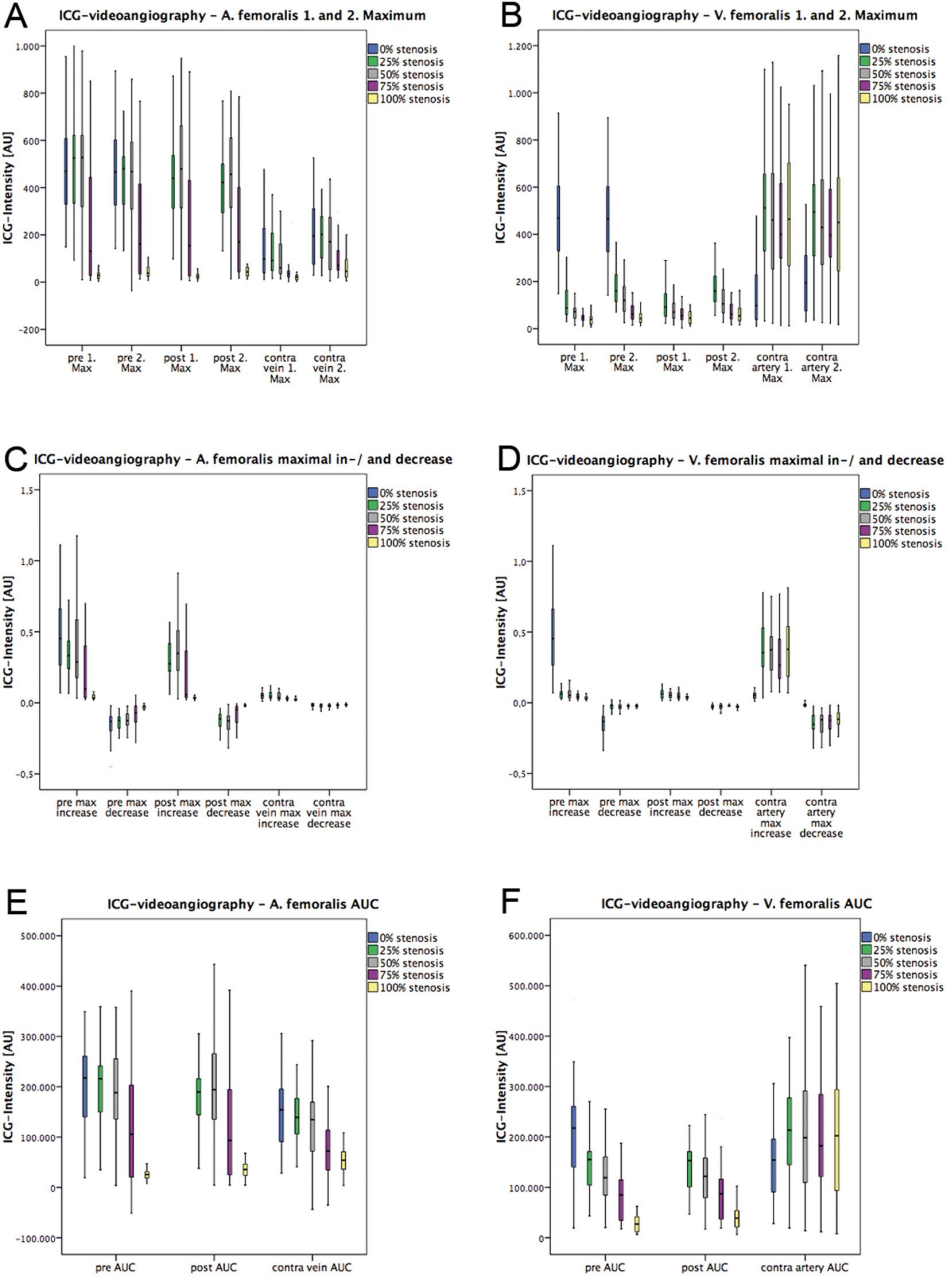
Descriptive results of indocyanine green-videoangiography (ICG) with integrated FLOW 800 tool. First and second maximum (**A,B**), maximal in-/decrease (**C**,**D**), and the area under the curve (AUC) (**E**,**F**) were analysed simultaneously at three regions of interest (ROI) (pre- and poststenotic, and accompanying artery/vein) at varying degrees of stenosis at the A. and V. femoralis. ICG intensity was registered for each parameter in arbitrary units [AU].

A total of 20 rats were operated successfully on both hind legs (left and right) and all animals survived the operation. All animals tolerated the intravenous injection of ICG well and no allergic reactions occurred. The median values with range (minimum-maximum) of the assessed parameters are given in Supplementary Tables S1–4. For illustration, the results are displayed as Boxplots in Figs. 1 and 2. A total of 520 flowmeter measurements were performed and 520 ROIs in ICG-videoangiography with corresponding FLOW 800 analyses have been set and analysed in 40 A. and V. femoralis and hind legs.

Flowmeter analyses with varying arterial or venous stenotic situations of the A. or V. femoralis showed at all three areas of measurements (pre-, poststenotic and accompanying A. or V. femoralis) an almost linear decrease of blood flow (Supplementary Table S1 and Fig. 1A,B). Median (range) arterial prestenotic values for 0%, 25%, 50%, 75% and 100% degrees of stenosis at the A. femoralis were 1.86 ml/min (0.42–3.24), 1.39 ml/min (0.20–2.94), 0.92 ml/min (0.22–2.74), 0.36 ml/min (0.08–1.66) and 0.00 ml/min (0.00–0.34), respectively. Median (range) arterial poststenotic values for 25%, 50%, 75% and 100% degrees of stenosis at the A. femoralis were 1.18 ml/min (0.26–2.76), 0.70 ml/min (0.14–2.44), 0.20 ml/min (0.04–1.40) and 0.00 ml/min (0.00–0.30), respectively. Median (range) venous prestenotic values for 0%, 25%, 50%, 75% and 100% degrees of stenosis at the V. femoralis were 1.58 ml/min (0.46–2.86), 1.22 ml/min (0.20–2.94), 0.85 ml/min (0.14–2.62), 0.29 ml/min (0.06–1.32) and 0.01 ml/min (0.0–0.7), respectively. Median (range) venous poststenotic values for 25%, 50%, 75% and 100% degrees of stenosis at the V. femoralis were 1.05 ml/min (0.20–2.78), 0.69 ml/min (0.10–2.48), 0.21 ml/min (0.04–1.28) and 0.01 ml/min (0.00–0.36), respectively.

Simultaneous laser-Doppler flowmetry and tissue spectrophotometry (O2C) measurements showed a decrease of all variables in superficial (S) and deep (D) detection planes in cases of increasing stenosis of the A. femoralis. SO_2_ D and Hb D showed an increase of values in cases of stenosis of the V. femoralis (58.0 AU (21.0–88.0) to 64.0 AU (0.0–96.0) and (70.5 AU (36.0–118.0) to 76.0 AU (32.0–124.0), respectively). All other variables showed a decrease in superficial and deep detection planes (Supplementary Table S2 and Fig. 1C–F).

The quantitative analysis of ICG-videoangiography showed an acute decrease of all variables (1^st^ and 2^nd^ maximum, maximal in- and decrease, and area under the curve (AUC)) at 75% and 100% degrees of stenosis at the A. femoralis in the prestenotic ROIs. In the poststenotic ROIs a valley of all variables was recognized at 25% degrees of stenosis at the A. femoralis. After 75% degrees of stenosis at the A. femoralis and more a continuous decrease of all variables was seen, but 0 arbitrary units [AU] was never reached at any degree of stenosis. At 25% degrees of stenosis at the A. femoralis the values for all variables showed an increase, followed by a nearly linear decrease in the corresponding V. femoralis (Supplementary Table S3 and Fig. 2).

All pre- and poststenotic variables showed a continuous decrease at 50% and 75% degrees of stenosis at the V. femoralis after a slight increase at 25% degrees of stenosis. At 100% degrees of stenosis at the V. femoralis an increase of 1^st^ and 2^nd^ maximum was seen. ICG-videoangiography AUC showed a continuous decrease at 50–100% stenosis of the V. femoralis. All variables in the corresponding A. femoralis were comparable to the control measurement without any stenosis (0%) at any degree of stenosis of the V. femoralis (Supplementary Table S4 and Fig. 2).

In summary, Tables 1 and 2 show the correlation coefficients according to Spearman in an analysis between the arterial and venous degree of stenosis and the analysed parameter for each modality.

**Table 1:** Correlation coefficients according to Spearman in an analysis between the degree of arterial stenosis and the analysed parameter for each modality (flowmeter, O2C and ICG-videoangiography).

Parameter	Estimate	p-value	Parameter	Estimate	p-value
FlowArtpre	−0.817	<0.001	ICGArtPre1Max	−0.559	<0.001
FlowArtPost	−0.842	<0.001	ICGArtPre2Max	−0.565	<0.001
FlowArtContra	−0.703	<0.001	ICGArtPreMaxincr	−0.548	<0.001
			ICGArtPreMaxdecr	0.495	<0.001
			ICGArtPreAUC	−0.545	<0.001
			ICGArtPost1Max	−0.594	<0.001
			ICGArtPost2Max	−0.568	<0.001
O2CArtSo2S	−0.328	<0.001	ICGArtPostMaxincr	−0.485	<0.001
O2CArtSo2D	−0.120	0.091	ICGArtPostMaxdecr	0.589	<0.001
O2CArtHbS	−0.252	0.000	ICGArtPostAUC	−0.540	<0.001
O2CArtHbD	0.010	0.887	ICGContra1Max	−0.464	<0.001
O2CArtVeloS	−0.417	<0.001	ICGContra2Max	−0.392	<0.001
O2CArtVeloD	−0.520	<0.001	ICGContraMaxincr	−0.320	<0.001
O2CArtFlowS	−0.398	<0.001	ICGCMaxdecr	0.115	0.111
O2CArtFlowD	−0.488	<0.001	ICGContraAUC	−0.440	<0.001

Abbreviations: flow = flowmeter; O2C = laser-Doppler flowmetry and tissue spectrophotometry; ICG = indocyanine green Coding key: Modality (flow, O2C or ICG) + vessel (artery) + localization (pre-, poststenotic or accompanying) + parameter.

**Table 2:** Correlation coefficients according to Spearman in a analysis between the degree of venous stenosis and the analysed parameter for each modality (flowmeter, O2C and ICG-videoangiography).

Parameter	Estimate	p-value	Parameter	Estimate	p-value
FlowVenpre	−0.811	<0.001	ICGVenPre1Max	−0.636	<0.001
FlowVenPost	−0.792	<0.001	ICGVenPre2Max	−0.702	<0.001
FlowVenContra	−0.529	<0.001	ICGVenPreMaxincr	−0.549	1.169
			ICGVenPreMaxdecr	0.471	<0.001
			ICGVenPreAUC	−0.656	<0.001
			ICGVenPost1Max	−0.269	0.001
			ICGVenPost2Max	−0.443	<0.001
O2CVenSo2S	−0.151	0.033	ICGVenPostMaxincr	−0.129	0.109
O2CVenSo2D	0.115	0.104	ICGVenPostMaxdecr	−0.004	0.959
O2CVenHbS	−0.216	0.002	ICGVenPostAUC	−0.532	<0.001
O2CVenHbD	0.146	0.038	ICGContra1Max	0.383	<0.001
O2CVenVeloS	−0.068	0.336	ICGContra2Max	0.297	<0.001
O2CVenVeloD	−0.169	0.017	ICGContraMaxincr	0.466	<0.001
O2CVenFlowS	−0.074	0.301	ICGContraMaxdecr	−0.386	<0.001
O2CVenFlowD	−0.173	0.015	ICGContraAUC	0.134	0.062

Abbreviations: flow = flowmeter; O2C = laser-Doppler flowmetry and tissue spectrophotometry; ICG = indocyanine green; Coding key: Modality (flow, O2C or ICG) + vessel (artery) + localization (pre-, poststenotic or accompanying) + parameter

### Diagnosis of stenotic condition

**Figure 3:**
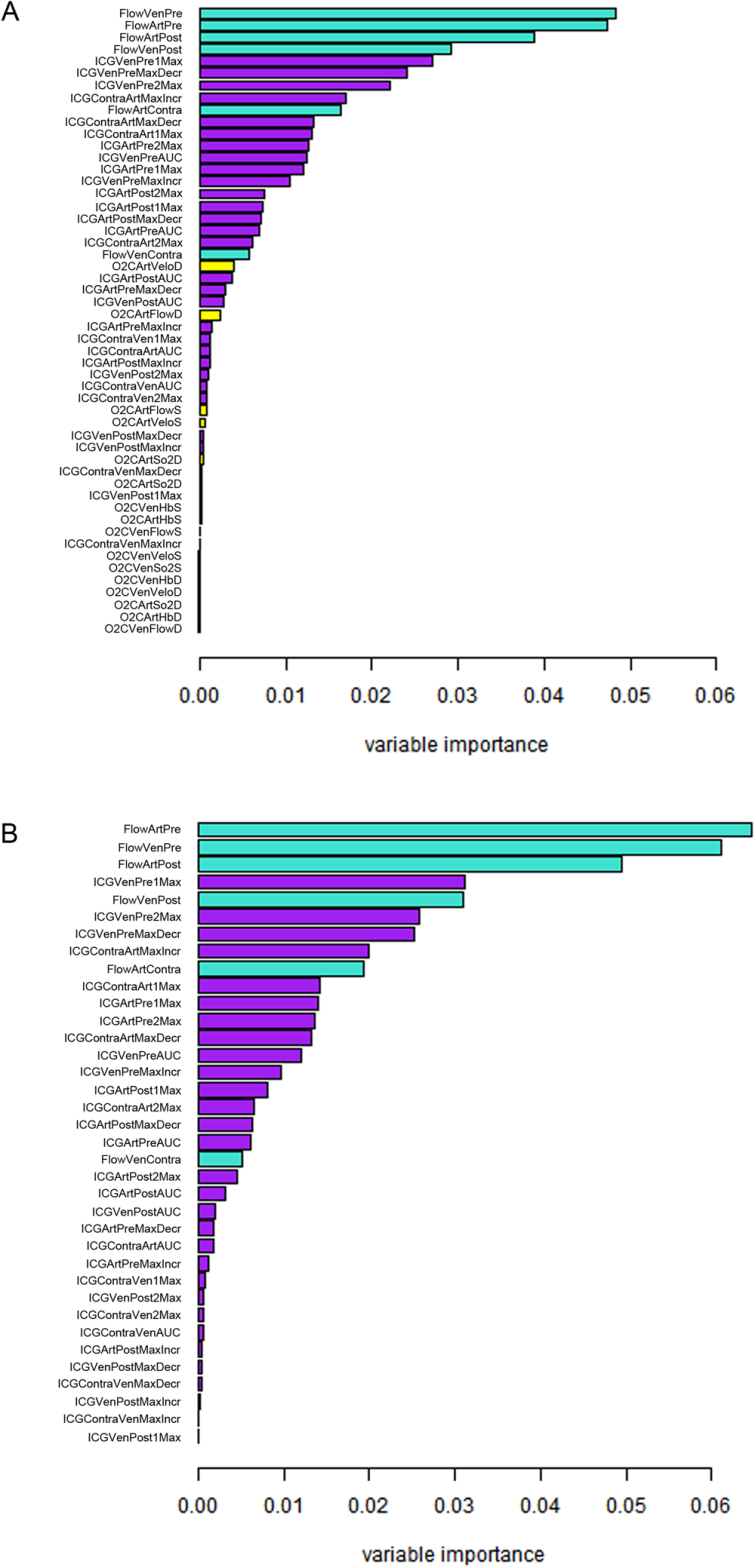
Random forest variable importance measures in (**A**) the full and (**B**) reduced model. A variable’s importance is measured by the difference of the model’s prediction accuracy (=relative frequency of correct classifications) before and after random permutation of a variable’s values. The permutation is used to nullify any relations of a variable to the outcome or other variables. The prediction accuracy stays unaffected by permutation of a variable if it is not of relevance for prediction. The variable importance takes a value of zero (or small negative values resulting from random variation) is such a case.

**Figure 4:**
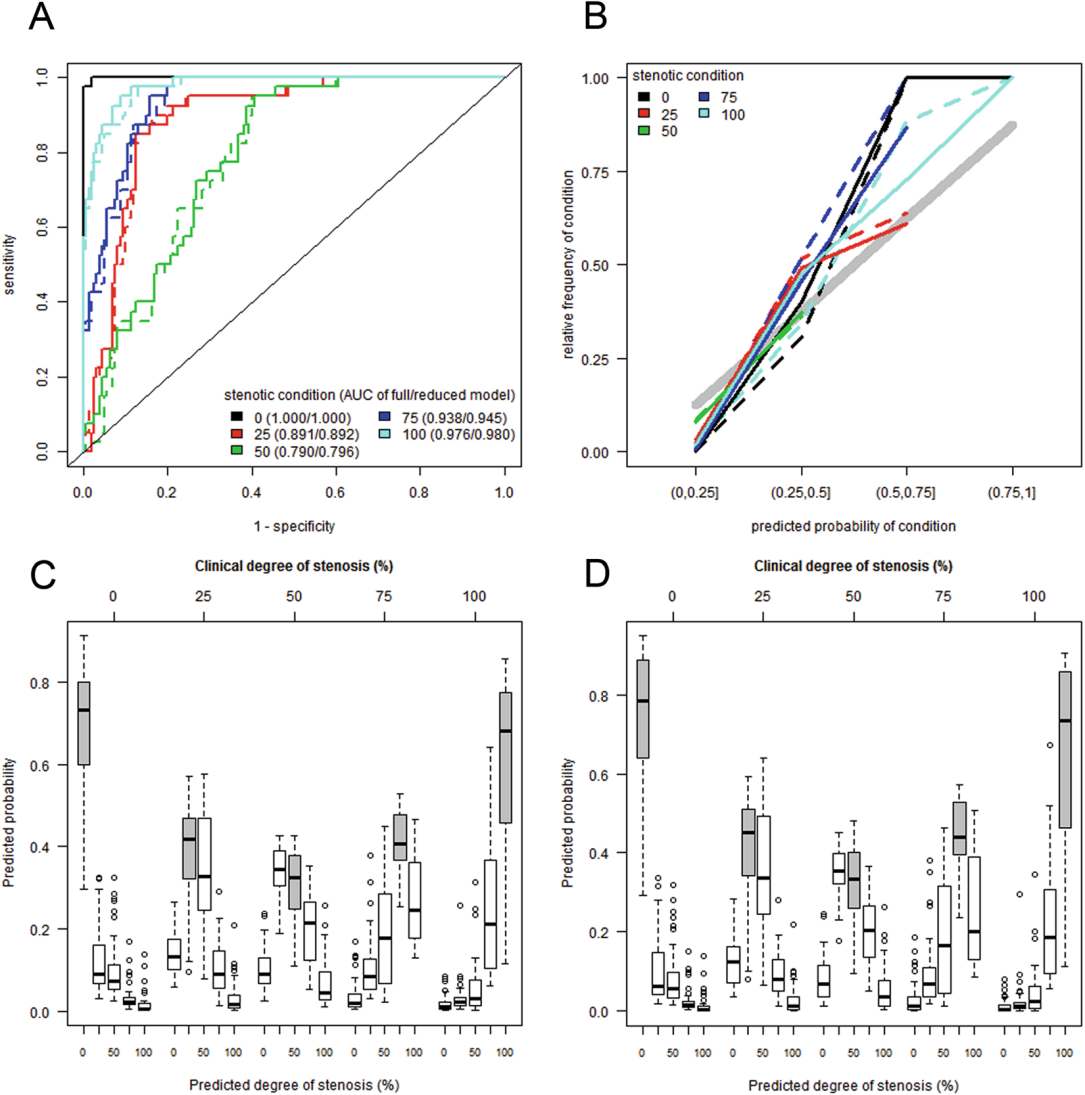
Model discrimination of named stenotic condition against all other conditions by Receiver Operating Characteristic (ROC) curve analysis and model calibration. Dashed and solid lines correspond to the full and reduced models, respectively. (**A**) The ROC curve is a plot of pairs of estimated sensitivity and 1-specificity resulting from the application of different cut-off values to the model’s predicted class probabilities. The area under the ROC (AUROC) curve serves as a summary measure of a model’s discriminatory ability across all possible cut-off values. Random guessing of classes relates to a value of 0.5 while perfect discrimination relates to a value of 1. (**B**) Good discrimination does not yield accurate prediction probabilities. Therefore, the predicted class probabilities (summarized by intervals) have to equal the expected frequencies (estimated by observed relative frequencies) for a well-calibrated prediction model. Distribution of probabilities predicted by the Random Forest model (y-axis) for each possible degree of stenosis (x-axis), grouped by the true clinical degree of stenosis (top axis) for the full (**C**) and reduced model (**D**). For example, stenoses with a true degree of 0% (=0 at top axis) are given the highest likelihoods (y-axis) to be of level 0% (x-axis) using the input parameters for prediction by the Random Forest model. Grey boxplots refer to the probabilities of predicting the true clinical degree of stenosis.

The variable importance measures of all three modalities used in the Random Forests prediction model showed a predominant importance of flowmeter and ICG-videoangiography analyses, thereby suggesting the exclusion of O2C measurements from the full model (Fig. 3A). The ranking of importance measures of flowmeter and ICG-videoangiography analyses stayed mainly unaffected in such a reduced model (Fig. 3B). The discrimination and calibration, measured by the area under the ROC curves (AUROC) and calibration plots, even slightly improved for the reduced model by the omission of O2C measurements from the full model (Fig. 4A,B). In summary, no stenosis and 100% degrees of stenosis were best predicted (AUROC 0.999 and 0.980 respectively) by the reduced model. Most difficult prediction remained a stenosis of 50%, which still resulted in a high AUROC of 0.801 (Fig. 4A). The calibration plots show a considerable agreement between predicted probabilities and relative frequencies of stenotic conditions (Fig. 4B). The Random Forest prediction model takes the flowmeter, the ICG-videoangiography and the O2C measurements as input and returns the likelihood that a stenosis is of degree 0%, 25%, …, or 100% as output. The distributions of these estimated likelihoods are presented in Fig. 4C,D, grouped by the actually observed level of stenosis. With a true level of 0%, for example, the stenoses are most likely classified as 0% by the prediction model. With a true level of 25% or 50% however, the prediction model returns equally high likelihoods for a classification into both of these levels. These results provide a more in-depth exploration of the discriminatory power of the models than the ROC analysis. The latter produced high AUC values as the classes 25% and 50% could still be very well discriminated from the other classes. The differentiation between 25% and 50% is more difficult though, which is shown by Fig. 4C (full model) and 4D (reduced model). Cohen’s (weighted) Kappa was computed in an additional analysis to measure agreement between observed and predicted stenotic conditions. It reached values of Κ = 0.815 and Κ = 0.823 for the full and reduced model, respectively (Table 3).

**Table 3:** Cross-classified table.

		Predicted degree of stenosis				
		0%	25%	50%	75%	100%
Clinical degree of stenosis	0%	**40**	0	0	0	0
	25%	1	**28**	9	2	0
	50%	1	17	**12 (13)**	10 (9)	0
	75%	0	1 (0)	2	**31 (33)**	6 (5)
	100%	0	0	0	7 (8)	**33 (32)**

Cross-table of observed vs. predicted stenotic conditions of the full model and the reduced model (given in brackets). Agreement was measured by Cohen’s weighted Kappa resulting to Κ = 0.815 (Κ = 0.823).

## Discussion

The current study analysed the quantitative and qualitative results, as well as the importance and validity of three established techniques in perfusion analyses for detection of different degrees of venous and arterial stenoses of the nourishing vessel in an established rat hind leg model^19^. Vascular compromise is likely to progress from partial to full obstruction (e.g. thrombosis and hematoma formation). In this process the time course may differ. In times of increasingly elderly and ill (ASA > II) patients needing local or free flap surgery^2^, early, reliable, reproducible and correct monitoring techniques are needed. Nevertheless, only a minority of studies have addressed the effect of partial to full occlusion of vascular supply and on the effectiveness of its detection in a methodically comparative study.

The technique for ICG-videoangiography as well as for O2C measurements are described elsewhere in detail^8,20–22^. The technical and clinical advantages of objective monitoring methods are generally obvious and well described, but most studies are focussing on the qualitative value of visualization of patency of microvascular anastomosis or tissue perfusion alone^14,23^. While most other studies focus on qualitative observations, we included a highly sophisticated statistical analysis based on machine learning (Random Forest prediction model) to compare the importance of each individual parameter that was quantitatively registered by each analysed modality. What is more, not two but three different modalities were analysed in this study. Interestingly, our results suggest that applying all three modalities for monitoring and detection of varying stenotic conditions was not superior to the combination of flowmeter and ICG-videoangiography alone (Figs. 3 and 4). While O2C data correctly predict the trend towards an increasing grade of stenosis, especially venous stenosis showed less significant correlation between degree of stenosis and registered parameter.

In detail, the simple flowmeter analyses showed interestingly the highest importance in the Random Forest prediction model. As described earlier, the combination with ICG-videoangiography can increase the validity of the assessment^17^. But as stated by Nasser *et al*. the detection of stenotic conditions is difficult. They reported significant fluorescence changes at 85% stenosis using the LifeCell SPY-Elite (LifeCell) system^19^. In contrast, we used the integrated software tool FLOW 800. With that, an initial drop of 1^st^ and 2^nd^ maxima were registered already with stenoses of 50% degrees. Our ROC curve analyses gave interesting insights, how validly each stenotic condition was detected. The results revealed that it was most difficult to correctly differentiate a stenosis of 50% degrees from the other stenotic degrees (Fig. 4A,C,D). Nevertheless, area under the ROC curve (AUROC) was 0.801 (without O2C), representing a high diagnostic power. AUROC for 25% and 75% degrees of stenosis were even higher (without O2C 0.892 and 0.943, respectively). So, in contrast to Nasser *et al*., even 25% degree of stenosis were correctly diagnosed with a high diagnostic power using a combination of flowmeter and ICG-videoangiography. Clinically ≥75% degrees of stenosis may begin to become relevant for distal organ perfusion, function and survival. Yang *et al*. described a more turbulent blood flow with a consecutively increased risk of distal embolization in a stenotic middle cerebral artery model^24^. Schoenberg *et al*. described a mean flow reduction of 50% at a stenotic degree of 90% at the renal artery^25^. A decrease of blood flow was also described by Nasser *et al*. In the presented study, the results of the flowmeter had the highest importance in the Random Forest prediction model. Further, also the parameters “*O2CVelo or O2CFlow*” had the highest importance in the Random Forest prediction model within the O2C measurements. These observations made by us and others reflect the significance of blood flow behavior itself in the difficult assessment of the (stenotic) vessel. Therefore, its valid detection is of great importance. Long-term effects were not analysed in this study, since the immediate detection and effects on vascular supply and flap physiology were of main interest.

Further, ICG-videoangiography in venous conditions showed also good results with good importance in the Random Forest prediction model, indicated by the values “*ICGVenPre1Max, ICGVenPre2Max and ICGVenPreMaxdecr”*. This is clinically of great importance, because venous congestion remains to be the leading reason for flap failure^26^. Early detection of flaps prone to fail is mandatory for successful flap salvage within a tissue/flap-dependent critical time of congestion, since blood flow changes occur earlier than clinical signs appear^6,27,28^. What is more, flap failure does not seem to be an “all or none phenomenon” as described by Weinzweig *et al*.^29^. They described the occurrence of slow flap failure as a result of microcirculatory disturbances due to persistent microthrombembolism. In this setting, the correct and valid detection of stenotic disease, that might facilitate microthrombembolism, is as crucial as a technically correct performed microvascular anastomosis.

In this context, the direct methods analysing blood flow changes in the pre- and post-stenotic area showed to be superior, in comparison to methods that are applied distally at the transplant´s surface (as seen in the O2C measurements in our study). Further, a single measurement is not enough and multiple measurements are needed to estimate the course more correctly while applying O2C^30^. In conclusion of the results for O2C measurements, the physiologic reaction on blood flow compromising situations results in delayed and hardly recognizable, and distinguishable changes in oxygen saturation, haemoglobin level, velocity and blood flow. Therefore, prediction of the clinical course may be limited though^31^. On the other hand, reliable and valid surface-based methods would be easier to use on the ward or intensive care unit and add an important, objective method to common clinical observation and tests including recapilarisation time, pinprick testing and surgical APGAR score^7,32^.

Akita *et al*. described a promising approach to increase the predictability of vascular nourishing of free flaps on the ward. They combined regional oxygen saturation monitoring with ICG-angiography. The regional oxygen saturation sensor was placed onto an intraoperatively localised ICG angiographically early-staining area^33^. With that Akita *et al*. described an AUROC of 1.0 for their developed oxygen saturation index. This result was further superior to simultaneously registered blood glucose measurement index.

On the basis of our results we will advocate a combination of methods as the safest way for reliable monitoring of critical and/or buried flaps. We are aware that our good results were reduced by the O2C measurements, but clinically the application of invasive methods as ICG-videoangiography or flowmeter analysis at the anastomotic region is not always immediately possible. Implantable Cook-Schwartz Doppler probes or flow coupler devices might be therefore the regional methods of choice in daily usage and improve salvage rates^9,34^. Moreover, a combination of two established methods is enough and an additional third method is not always beneficial.

The presented study is based on a small animal model. Potential translation to clinical care is controversial and therefore our results have to be interpreted with caution. Nevertheless, rats remain the gold standard in the field of microsurgical research^35^ and during a time of recurrent ethical questions and surveys, preclinical studies still have to be performed.

The main disadvantage of ICG (video-) angiography is its cost, especially in the case of an integrated system in an operating microscope, as previously described by others^36^. Based on our experience, the possibility to decrease free flap failure and to increase salvage rates outweigh the perceived costs. Further, we only described values for the OPMI Pentero integrated near-infrared videoangiography detection system with the integrated FLOW 800 tool (INFRARED 800; Carl Zeiss Meditec AG; Oberkochen, Germany); for other systems, results may differ. The next logical step from this work, will be the analysis of the degree of vascular stenosis. A study in a larger animal model with different types of composite flaps would be necessary to increase the clinical relevance and translation to clinical care.

## Methods

### Ethical statement

The study conformed with current German regulations, with guidelines for animal welfare, and to the international principles of laboratory animal care. The local government approved the animal experiments (Regierung Oberbayern; AZ.: 55.2-1-54-2532-85-15). The animals were housed in filter-top cages under hygienic conditions according to the guidelines of the Federation of Laboratory Animal Science Associations (FELASA). Water and standard rodent diet (Altromin; Altromin Spezialfutter GmbH & Co. KG; Lage, Germany) were given ad libitum. The operations were final experiments and no postoperative visits were necessary.

### Operation

Male Wistar rats weighing between 300 and 350 g were initially anesthetized with intraperitoneal administration of ketamine 10% (1 mL/kg/weight) and xylazine 2% (0.25 mL/kg/weight) supplemented by intravenous application of one-eighth doses 10% ketamine when needed using an inserted venous microcatheter (Premicath; VYGON GmbH & Co. KG; Aachen, Germany) in the external jugular vein according to the femoral vein access as previously described^37,38^.

**Figure 5:**
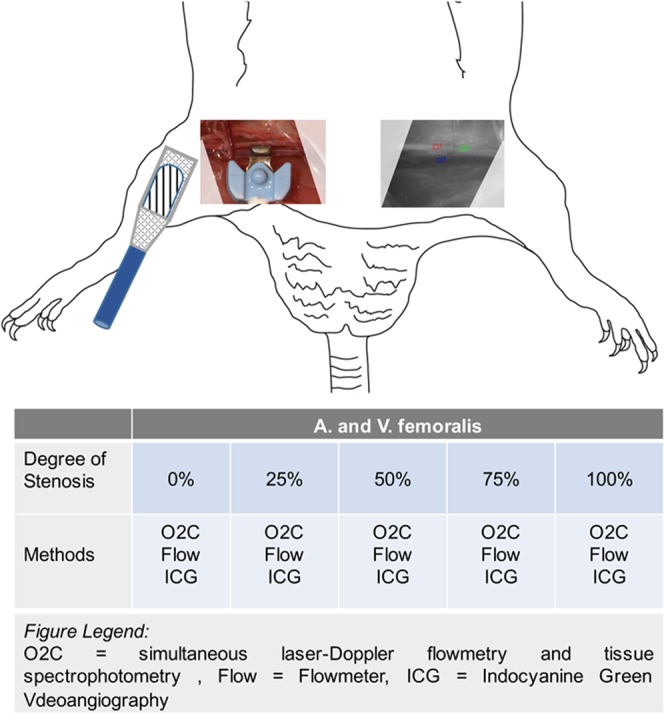
Experimental setup in the hind leg model of the rat. Flowmeter, simultaneous laser-Doppler flowmetry and tissue spectrophotometry and indocyanine green-videoangiography with integrated FLOW 800 tool were used to detect different degrees of stenotic situations at the A. and V. femoralis (0%, 25%, 50%, 75% and 100%) and analyse the consecutive blood flow changes.

All 20 rats were operated in a standardized fashion. The inguinal region and the hind legs on both sides were shaved. After a sharp inguinal incision of the skin and subcutaneous tissue, the femoral neurovascular sheath (femoral artery, vein and nerve) was exposed and the vessels were circumferentially prepared. All vascular branches between the inguinal ligament and epigastric vessels were ligated. Following increasing degrees of arterial and venous stenosis (each 0%, 25%, 50%, 75% and 100%) were applied separately by tightening a perivascular suture under visual control with millimetre gauge (Fig. 5)^17^. After each step of vascular stenosis different flow measurements and analyses (flowmeter, simultaneous laser-Doppler flowmetry and tissue spectrophotometry and ICG-videoangiography with integrated FLOW 800 tool) were performed.

### Analyses

After arterial or venous stenosis (0%, 25%, 50%, 75% or 100%) has been set, ten minutes were waited to establish a regular and adapted flow. Following that, blood flow was measured pre-, post-stenotic and at the accompanying arterial or venous vessel for 30 seconds with a flowmeter (TS-420; Transonic System Inc.; Ithaca, NY, USA). An average of five measurements within these 30 seconds was taken as the result, in order to reduce a bias due to movement or spasm during measurement. After flowmetry, intraoperative ICG-videoangiography of the femoral vein and artery were performed using the operating microscope type OPMI Pentero with integrated near-infrared videoangiography detection system and FLOW 800 tool (INFRARED 800; Carl Zeiss Meditec AG; Oberkochen, Germany)^17,39^. The ICG (ICG-PULSION; Pulsion Medical System AG; Munich, Germany) was injected (0.3 mg/kg body weight, 25 mg dissolved in 5 ml sterile water) as a bolus into the external jugular vein using the inserted venous microcatheter (Premicath; VYGON GmbH & Co.KG; Aachen, Germany). The ICG-videoangiography was conducted at a fixed working distance of 300 mm and with a 5-fold magnification. Registration of the emission signal was started immediately after injection of the dye and was recorded for a period of 120 seconds with 25 images per second. All data were immediately analysed with a mathematical software tool FLOW 800 (FLOW 800; Carl Zeiss AG; Oberkochen, Germany) and colour encoded with respect to fluorescence over time. The fluorescence intensity was recorded as arbitrary units [AU]^21^. In cases of 0% stenosis only two regions of interest (ROI) were positioned, one in the femoral vein and one in the femoral artery (number of ROIs for 0% stenosis n = 2). In all other stenotic degrees (25%, 50%, 75% and 100%) three ROIs were positioned: pre-, poststenotic and at the accompanying artery or vein. For each ROI 5 defined parameter were registered: 1. and 2. Maximum, maximal increase and decrease, area under the curve for a period of 120 seconds.

Finally, simultaneous laser-Doppler flowmetry and tissue spectrophotometry of the ipsilateral hind leg (O2C; LEA Medizintechnik GmbH; Giessen, Deutschland) was performed^8^. For the measurement, the skin was cleaned and dried and the O2C sensor was positioned in the centrally of the ipsilateral hind leg (Fig. 5). The measurement was performed for 30 seconds to reduce bias due to movements. With one measurement oxygen saturation (SO_2_, in arbitrary units, [AU]), haemoglobin level (Hb, in [AU]), velocity (velo, in [AU]) and blood flow (flow, in [AU]) were non-invasively calculated superficially at the skin surface (S) and at a depth (D) of 8 mm. The rat was sacrificed in deep anaesthesia with intracardiac injection of pentobarbital 60 mg/kg body weight (Narcoren, Fa. Rhone Merieux GmbH, Laupheim) following a standard protocol^40^ after all measurements on both hind legs have been performed.

Following coding key was used for illustration in Fig. 3 and Tables 2 and 3: modality (flow, O2C or ICG) + vessel (stenosis at femoral artery or vein) + localization of flowmeter probe or ROIs in ICG-videoangiography (pre-, poststenotic or accompanying) + parameter.

### Statistical methods

Study data were prospectively collected and analysed. The distribution of continuous data is presented by median (range) and illustrated by boxplots. Computations have been performed using IBM SPSS Statistics for Windows, version 23.0 (IBM Corp., Armonk, New York, USA). Machine Learning was used to enable a flexible modelling of the potentially complex and interacting relations between flowmeter, ICG-videoangiography and O2C measurements to stenotic conditions. Random Forests based on conditional inference trees provide a corresponding model structure, ensure unbiased variable selection and are well-known for their outstanding prediction performance^41^. As each tree in a Random Forest is fit to a random sample of the data, the left-out observations can be used for an unbiased assessment of performance measures and model diagnostics. Additional test data for validation was therefore not necessary in the present investigations. Variable importance measures were computed to explore the relevance of flowmeter, ICG-videoangiography and O2C measurements for the diagnosis of stenotic conditions^42^. The discriminatory and diagnostic ability of the prediction model was assessed by the area under the curve (AUROC) of the Receiver Operating Characteristic (ROC) curve. Calibration plots were used to investigate the agreement of predicted probabilities and observed relative frequencies of stenotic conditions. In similarity to that, the agreement of observed and predicted stenotic conditions was quantified by Cohen’s Kappa. The Random Forests model consisted of 5000 trees to achieve stability of results and to increase prediction performance. It was fit to the data using R 3.5.0 (R Foundation for Statistical Computing, Vienna, Austria) and the function cforest(), leaving all meta parameters of the function, except for the number of trees, unchanged^41^. The degrees of stenosis served as multinomial outcome. The predictor variables of the model are listed in Fig. 3.

## Supplementary information

Supplementary Figures S1-2.

Supplementary Tables S1-4.

## Data Availability

All data generated or analyzed during this study are included in this published article (and its Supplementary Information files).
