# Publisher Correction: A comparative analysis using flowmeter, laser-Doppler spectrophotometry, and indocyanine green-videoangiography for detection of vascular stenosis in free flaps

**DOI:** 10.1038/s41598-020-60910-w

**Published:** 2020-02-28

**Authors:** Thomas Mücke, Alexander Hapfelmeier, Leonard H. Schmidt, Andreas M. Fichter, Anastasios Kanatas, Klaus-Dietrich Wolff, Lucas M. Ritschl

**Affiliations:** 1 Department of Oral and Maxillofacial Surgery, Malteser Kliniken Rhein-Ruhr, Krefeld-Uerdingen, Germany; 2 Institute of Medical Informatics, Statistics and Epidemiology, School of Medicine, Technical University of Munich, Munich, Germany; 3 Department of Oral and Maxillofacial Surgery, School of Medicine, Technical University of Munich, Klinikum rechts der Isar, Munich, Germany; 4 Leeds Teaching Hospitals, St James Institute of Oncology and Leeds Dental Institute, Munich, Germany

Correction to: *Scientific Reports* 10.1038/s41598-020-57777-2, published online 22 January 2020

The original version of this Article contained an error in the title of the paper, where the word “spectrophotometry” was incorrectly given as “|spectrophotometry”. This has now been corrected in the PDF and HTML versions of the Article.

