# Supplementary material for: A comparative analysis using flowmeter, laser-Doppler |spectrophotometry, and indocyanine green-videoangiography for detection of vascular stenosis in free flaps
Source: Sci Rep. 2020 Jan 22;10:939. doi: 10.1038/s41598-020-57777-2 (PMC6976589; doi:10.1038/s41598-020-57777-2)
Supplement: Supplementary file 1 — Supplementary Figures S1-2. [file 41598_2020_57777_MOESM1_ESM.pdf]

Supplementary Information – Figures

A comparative analysis using flowmeter, laser-Doppler spectrophotometry, and indocyanine green-videoangiography for detection of vascular stenosis in free flaps

Thomas Mücke [#], [1] MD DDS PhD; Alexander Hapfelmeier [#], [2] PhD; Leonard H. Schmidt, [3] MD DMD; Andreas M. Fichter, [3] MD DDS PhD; Anastasios Kanatas, [4] MD PhD FDS FRCS; Klaus-Dietrich Wolff, [3] MD DDS PhD; Lucas M. Ritschl *, [3] MD DMD PhD

[#] both authors contributed equally to this paper

[1] Department of Oral and Maxillofacial Surgery, Malteser Kliniken Rhein-Ruhr, Krefeld-Uerdingen, Germany

[2] Institute of Medical Informatics, Statistics and Epidemiology, Technische Universität München, Germany

[3] Department of Oral and Maxillofacial Surgery, Klinikum rechts der Isar, Technische Universität München, Germany

[4] Leeds Teaching Hospitals, St James Institute of Oncology and Leeds Dental Institute

Supplementary Figure Legend

Supplementary Figure S1. ICG-videoangiography measurements with varying degrees of stenosis at the A. femoralis. Shows the intraoperative FLOW 800 analysis (first column). The second and third columns show the ICG-videoangiography in the arterial and venous phases respectively with defined three regions of Interest (ROIs). The non-parametric analysis of the measured intensity of ICG-videoangiography over time of the femoral vessels with varying degrees of arterial stenosis (4th column) are displayed in graphs (y-axis: ICG intensity [AU]; x-axis: time [s]) for each corresponding ROI.

Supplementary Figure S2. ICG-videoangiography measurements with varying degrees of stenosis at the V. femoralis. Shows the intraoperative FLOW 800 analysis (first column), ICG-videoangiography with included ROIs (second and third column). The second and third columns show the ICG-videoangiography in the arterial and venous phases respectively with defined three regions of Interest (ROIs). The non-parametric analysis of the measured intensity of ICG-videoangiography over time of the femoral vessels with varying degrees of venous stenosis (4th column) are displayed in graphs (y-axis: ICG intensity [AU]; x-axis: time [s]) for each corresponding ROI.

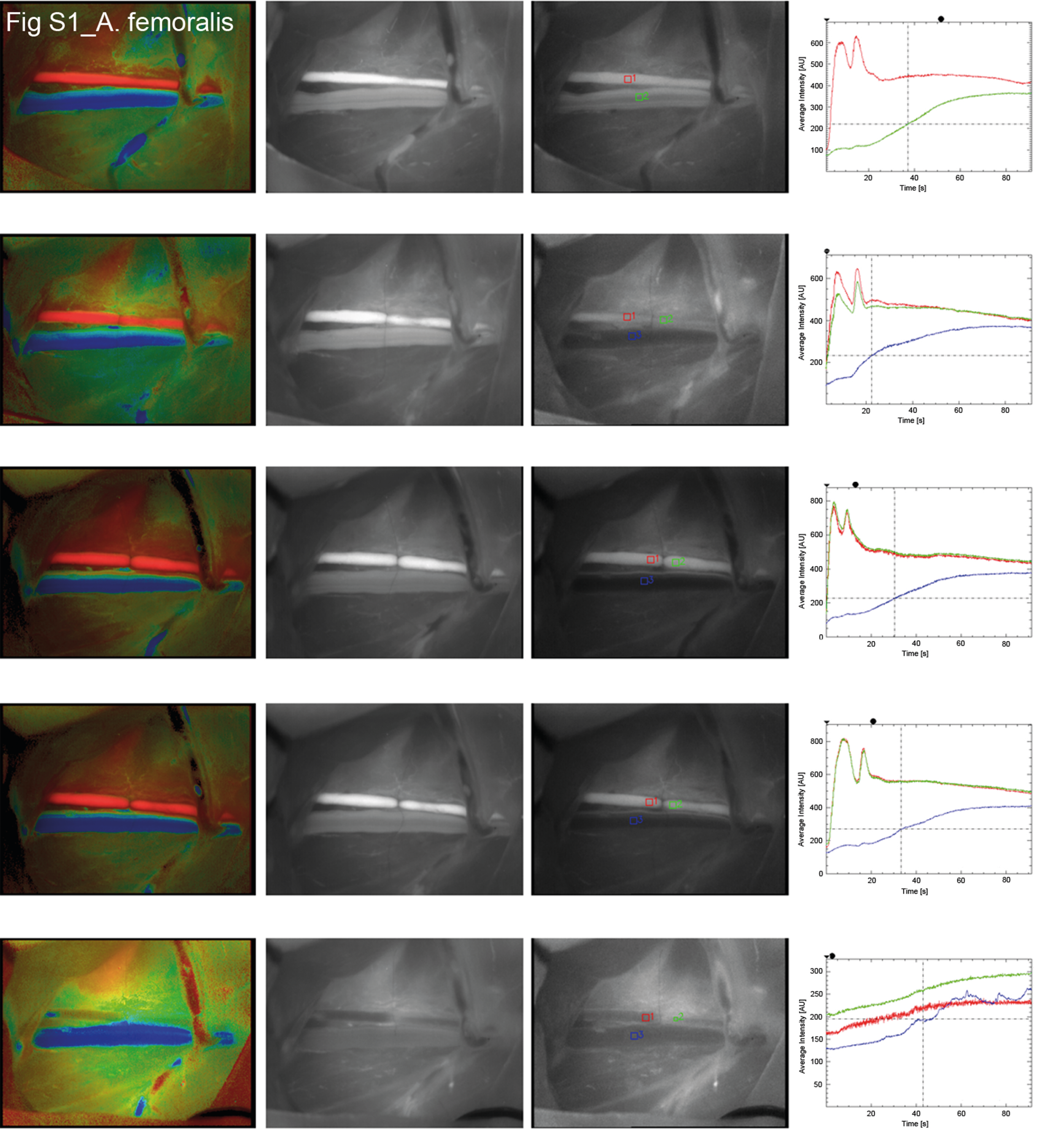
Fig S1_A. femoralis
Average Intensity [AU]
Time [s]
1
2
3

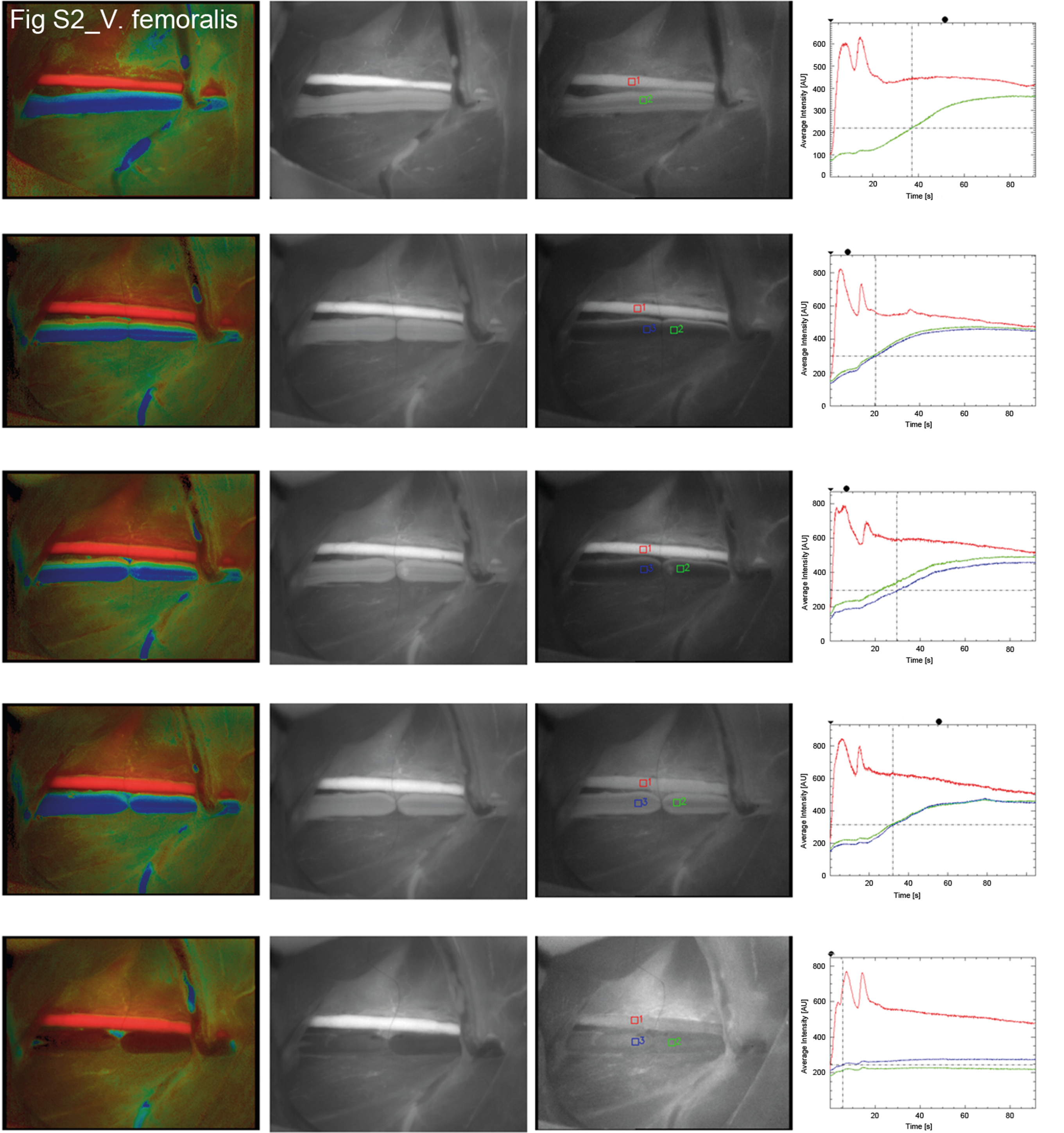

Fig S2_V. femoralis
