# Supplementary material for: A comparative analysis using flowmeter, laser-Doppler |spectrophotometry, and indocyanine green-videoangiography for detection of vascular stenosis in free flaps
Source: Sci Rep. 2020 Jan 22;10:939. doi: 10.1038/s41598-020-57777-2 (PMC6976589; doi:10.1038/s41598-020-57777-2)
Supplement: Supplementary file 2 — Supplementary Tables S1-4. [file 41598_2020_57777_MOESM2_ESM.pdf]

Supplementary data set – Supplementary Tables

A comparative analysis using flowmeter, laser-Doppler spectrophotometry, and indocyanine green-videoangiography for detection of vascular stenosis in free flaps

Thomas Mücke [#], [1] MD DDS PhD; Alexander Hapfelmeier [#], [2] PhD; Leonard H. Schmidt, [3] MD DMD; Andreas M. Fichter, [3] MD DDS PhD; Anastasios Kanatas, [4] MD PhD FDS FRCS; Klaus-Dietrich Wolff, [3] MD DDS PhD; Lucas M. Ritschl *, [3] MD DMD PhD

[#] both authors contributed equally to this paper

[1] Department of Oral and Maxillofacial Surgery, Malteser Kliniken Rhein-Ruhr, Krefeld-Uerdingen, Germany

[2] Institute of Medical Informatics, Statistics and Epidemiology, Technische Universität München, Germany

[3] Department of Oral and Maxillofacial Surgery, Klinikum rechts der Isar, Technische Universität München, Germany

[4] Leeds Teaching Hospitals, St James Institute of Oncology and Leeds Dental Institute

Supplementary Tables

Supplementary Table S1. The median (range) results of flowmeter analysis with varying stenotic situations at the A. or V. femoralis.

Supplementary Table S2. The median (range) perioperative results with simultaneous laser-Doppler flowmetry and tissue spectrophotometry (O2C) at varying stenotic situations of the A. or V. femoralis in arbitrary units.

Supplementary Table S3. The median (range) results of ICG-videoangiography with varying stenotic situations at the A. femoralis.

Supplementary Table S4. The median (range) results of ICG-videoangiography with varying stenotic situations at the V. femoralis.

Supplementary Table S1. The median (range) results of flowmeter analysis with varying stenotic situations at the A. or V. femoralis.

**Stenosis**	**Artery prestenotic [ml/min]**	**Artery poststenotic [ml/min]**	**V. Femoralis [ml/min]**
**0%**	1.86 (0.42-3.24)	/	1.58 (0.46-2.86)
**25%**	1.39 (0.2-2.94)	1.18 (0.26-2.76)	1.28 (0.26-2.34)
**50%**	0.92 (0.22-2.74)	0.7 (0.14-2.44)	1.0 (0.24-2.06)
**75%**	0.36 (0.08-1.66)	0.2 (0.04-1.4)	0.46 (0.08-2.56)
**100%**	0.0 (0.0-0.34)	0.0 (0.0-0.3)	0.13 (0-1.16)
**Stenosis**	**Vein prestenotic [ml/min]**	**Vein poststenotic [ml/min]**	**A. Femoralis [ml/min]**
**0%**	1.58 (0.46-2.86)	/	1.86 (0.42-3.24)
**25%**	1.22 (0.2-2.94)	1.05 (0.2-2.78)	1.46 (0.28-3.04)
**50%**	0.85 (0.14-2.62)	0.69 (0.1-2.48)	1.13 (0.26-2.98)
**75%**	0.29 (0.06-1.32)	0.21 (0.04-1.28)	0.85 (0.16-3.12)
**100%**	0.01 (0.0-0.7)	0.01 (0.0-0.36)	0.53 (0.08-2.34)

Supplementary Table S2. The median (range) perioperative results with simultaneous laser-Doppler flowmetry and tissue spectrophotometry (O2C) at varying stenotic situations of the A. or V. femoralis in arbitrary units.

Variable	Vessel	0%	25%	50%	75%	100%
**SO_2 S**	A. fem.	18.5 (1.0-55.0)	26.0 (1.0-60.0)	16.5 (1.0-54.0)	13.0 (1.0-54.0)	7.5 (0.0-68.0)
	V. fem.	18.5 (1.0-55.0)	19.5 (1.0-55.0)	18.5 (0.0-57.0)	15.0 (1.0-42.0)	16.0 (1.0-43.0)
**Hb S**	A. fem.	76.0 (51.0-89.0)	76.5 (55.0-93.0)	73.5 (61.0-90.0)	71.5 (52.0-103.0)	69.5 (58.0-93.0)
	V. fem.	76.0 (51.0-89.0)	69.0 (61.0-91.0)	70.0 (60.0-97.0)	71.0 (53.0-92.0)	69.5 (49.0-99.0)
**Velo S**	A. fem.	15.5 (9.0-51.0)	15.5 (7.0-63.0)	14.5 (6.0-55.0)	14.0 (2.0-48.0)	10.0 (1.0-28.0)
	V. fem.	15.5 (9.0-51.0)	18.0 (9.0-60.0)	22.0 (8.0-59.0)	15.5 (5.0-59.0)	15.5 (1.0-45.0)
**Flow S**	A. fem.	65.0 (4.0-309.0)	61.0 (5.0-284.0)	43.0 (2.0-231.0)	38.0 (1.0-187.0)	18.0 (1.0-132.0)
	V. fem.	65.0 (4.0-309.0)	60.0 (13.0-322.0)	74.5 (6.0-306.0)	56.5 (5.0-295.0)	50.5 (1.0-297.0)
**SO_2 D**	A. fem.	58.0 (21.0-88.0)	62.0 (45.0-96.0)	126.4 (40.0-84.0)	58.0 (19.0-73.0)	56.0 (0.0-87.0)
	V. fem.	58.0 (21.0-88.0)	62.5 (0.0-87.0)	64.5 (47.0-99.0)	65.0 (35.0-95.0)	64.0 (0.0-96.0)
**Hb D**	A. fem.	70.5 (36.0-118.0)	68.5 (45.0-149.0)	72.5 (49.0-137.0)	73.0 (51.0-108.0)	67.0 (46.0-101.0)
	V. fem.	70.5 (36.0-118.0)	70.5 (54.0-115.0)	72.0 (52.0-155.0)	73.0 (50.0-110.0)	76.0 (32.0-124.0)
**Velo D**	A. fem.	25.0 (10.0-54.0)	27.0 (10.0-63.0)	22.0 (10.0-55.0)	16.5 (7.0-45.0)	13.0 (6.0-22.0)
	V. fem.	25.0 (10.0-54.0)	26.0 (11.0-52.0)	26.0 (9.0-61.0)	23.5 (8.0-59.0)	22.0 (6.0-36.0)
**Flow D**	A. fem.	129.0 (28.0-269.0)	123.5 (28.0-278.0)	100.0 (25.0-267.0)	69.5 (6.0-252.0)	49.5 (4.0-118.0)

	V. fem.	129.0 (28.0-269.0)	120.0 (45.0-243.0)	126.0 (13.0-265.0)	107.0 (10.0-300.0)	96.0 (4.0-242.0)

Abbreviation: SO_2 = oxygen saturation; Hb = hemoglobin level; Velo = velocity; Flow = blood flow; S = superficial; D = deep; A./V. fem. = A. or V. femoralis

**Supplementary Table S3.** The median (range) results of ICG-videoangiography with varying stenotic situations at the A. femoralis.					
	**1. Max**	**2. Max**	**Max. increase**	**Max. decrease**	**AUC**
**0% A. fem.**	469.6 (148.3-955.5)	465.7 (142.2-894.3)	0.46 (0.07-4.64)	-0.13 (-0.43-(-0.02))	217,577 (19,490-478,345
**0% V. fem.**	98.1 (10.5-478.2)	194.6 (29.6-526.7)	0.05 (0.01-0.29)	-0.02 (-0.23-(-0.02))	154,149 (28,280-306,007)
**25% presten.**	517.5 (91.5-999.6)	480.8 (131.7-879.4)	0.35 (0.07-1.2)	-0.13 (-0.25-(-0.4))	215,930 (35,245-479,599)
**25% poststen.**	439.7 (98.1-872.8)	422.2 (132.1-767.5)	0.29 (0.06-0.89)	-0.12 (-0.31-(-0.0))	190,059 (31,886-425,727)
**25% V. fem.**	91.9 (15.6-572.5)	202.1 (27.5-634.7)	0.05 (0.01-0.26)	-0.02 (-0.16-(-0.01))	139,254 (40,976-392,915)
**50% presten.**	528.5 (10.6-978.9)	467.6 (0.0-859.5)	0.3 (0.03-1.27)	-0.14 (-0.35-(-0.03))	190,432 (0.0-456,781)
**50% poststen.**	479.3 (9.7-948.6)	434.3 (0.0-809.5)	0.35 (0.03-1.15)	-0.13 (-0.32-(-0.01))	188,260 (0.0-443,019)
**50% V. fem.**	62.0 (14.2-300.9)	172.3 (4.4-435.8)	0.04 (0.02-0.35)	-0.02 (-0.15-(-0.01))	134,757 (0.0-291,841)
**75% presten.**	130.6 (7.8-851.9)	161.2 (13.5-766.2)	0.1 (0.02-1.36)	-0.74 (-0.34-(-0.0))	103,051 (0.0-390,251)
**75% poststen.**	154.3 (5.6-890.4)	170.6 (16.9-785.2)	0.07 (0.02-1.31)	-0.05 (-0.25-(-0.01))	93,534 (4,546-391,633)
**75% V. fem.**	43.2 (1.4-450.9)	80.8 (18.5-560.1)	0.03 (0.01-0.71)	-0.02 (-0.1-(-0.0))	77,617 (0.0-358,659)
**100% presten.**	28.9 (3.6-614.9)	41.6 (7.3-625.4)	0.04 (0.02-3.55)	-0.03 (-0.24-(-0.0))	26,762 (0.0-250,227)
**100% poststen.**	28.1 (3.6-809.8)	51.3 (12.7-809.1)	0.03 (0.02-1.62)	-0.02 (-0.15-(-0.01))	37,925 (4,322-341,961)
**100% V. fem.**	21.8 (2.7-540.2)	64.9 (4.9-581.1)	0.02 (0.01-2.06)	-0.01 (-0.13-(-0.0))	55,725 (4,079-258,740)

Abbreviation:

Max. = Maximum or Maximal; A./V. fem. = A. or V. femoralis; presten. = prestenotic; poststen. = poststenotic; AUC = Area Under the Curve

Supplementary Table S4. The median (range) results of ICG-videoangiography with varying stenotic situations at the V. femoralis.					
	**1. Max**	**2. Max**	**Max. increase**	**Max. decrease**	**AUC**
**0% V. fem.**	98.1 (10.5-478.2)	194.6 (29.6-526.7)	0.05 (0.01-0.29)	-0.02 (-0.23-(-0.02))	154,149 (28,280-306,007)
**0% A. fem.**	469.6 (148.3-955.5)	465.7 (142.2-894.3)	0.46 (0.07-4.64)	-0.13 (-0.43-(-0.02))	217,577 (19,490-478,345
**25% presten.**	93.3 (30.2-497.8)	168.3 (69.9-529.8)	0.06 (0.02-0.82)	-0.02 (-0.18-0.0)	155,149 (43,254-363,928)
**25% poststen.**	96.2 (23.0-508.9)	172.7 (55.9-521.2)	0.07 (0.01-0.8)	-0.03 (-2.28-0.0)	156,840 (46,902-367,381)
**25% A. fem.**	512.1 (32.5-1,099.3)	494.9 (35.2-1,031.5)	0.38 (0.04-1.99)	-0.15 (-0.36-(-0.02))	213,213 (19,463-525,190)
**50% presten.**	81.1 (14.3-467.3)	129.3 (24.7-557.9)	0.05 (0.02-0.68)	-0.03 (-0.16-0.0)	119,085 (200,60-352,903)
**50% poststen.**	76.9 (14.4-461.1)	134.6 (25.7-511.2)	0.06 (0.02-0.81)	-0.03 (-0.13-0.0)	121,906 (17,044-344,468)
**50% A. fem.**	460.3 (22.4-1,129.6)	429.7 (25.8-1,093.4)	0.39 (0.08-1.43)	-0.13 (-0.47-(-0.04))	198,450 (13,707-540,864)
**75% presten.**	53.8 (10.0-496.2)	67.3 (14.9-526.3)	0.05 (0.01-0.89)	-0.03 (-0.13-(-0.01))	84,866 (17,485-187,371)
**75% poststen.**	55.6 (2.9-342.5)	75.5 (16.2-381.9)	0.05 (0.01-0.8)	-0.02 (-0.3-(-0.01))	87,259 (19,028-242,499)
**75% A. fem.**	415.2 (12.3-1,152.4)	404.6 (21.5-1,085.2)	0.29 (0.07-2.04)	-0.13 (-0.3-(-0.02))	182,372 (11,853-458,787)
**100% presten.**	40.6 (7.3-563.5)	47.9 (11.2-594.5)	0.05 (0.01-2.05)	- 0.02 (-0.1-(-0.01))	27,629 (6,832-390,336)
**100% poststen.**	45.7 (10.8-662.4)	62.8 (15.6-663.1)	0.04 (0.02-1.9)	-0.03 (-0.09-(-0.01))	41,172 (6,997-414,640)
**100% A. fem.**	464.1 (11.2-952.2)	450.2 (17.4-1,158.7)	0.38 (0.07-1.99)	-0.12 (-0.28-(-0.02))	202,315 (8,092-504,540)

Abbreviation:

Max. = Maximum or Maximal; A./V. fem. = A. or V. femoralis; presten. = prestenotic; poststen. = poststenotic; AUC = Area Under the Curve
